# Spatially Correlated, Single Nanomaterial-Level Structural and Optical Profiling of Cu-Doped ZnO Nanorods Synthesized via Multifunctional Silicides

**DOI:** 10.3390/nano8040222

**Published:** 2018-04-07

**Authors:** Johnson Truong, Matthew Hansen, Brian Szychowski, Tian Xie, Marie-Christine Daniel, Jong-in Hahm

**Affiliations:** 1 Department of Chemistry, Georgetown University, 37th & O Sts. NW., Washington, DC 20057, USA; jtt37@georgetown.edu (J.T.); mnh35@georgetown.edu (M.H.); tx19@georgetown.edu (T.X.); 2 Department of Chemistry and Biochemistry, University of Maryland Baltimore County, 1000 Hilltop Circle, Baltimore, MD 21250, USA; bszy1@umbc.edu (B.S.); mdaniel@umbc.edu (M.-C.D.)

**Keywords:** zinc oxide, copper-doped zinc oxide, copper zinc oxide, 1D nanomaterial, nanorod, luminescence, fluorescence, Raman scattering

## Abstract

We demonstrate a straightforward and effective method to synthesize vertically oriented, Cu-doped ZnO nanorods (NRs) using a novel multipurpose platform of copper silicide nanoblocks (Cu_3_Si NBs) preformed laterally in well-defined directions on Si. The use of the surface-organized Cu_3_Si NBs for ZnO NR growth successfully results in densely assembled Cu-doped ZnO NRs on each NB platform, whose overall structures resemble thick bristles on a brush head. We show that Cu_3_Si NBs can uniquely serve as a catalyst for ZnO NRs, a local dopant source of Cu, and a prepatterned guide to aid the local assembly of the NRs on the growth substrate. We also ascertain the crystalline structures, optical properties, and spectroscopic signatures of the Cu-doped ZnO NRs produced on the NBs, both at each module of NRs/NB and at their ensemble level. Subsequently, we determine their augmented properties relative to the pristine form of undoped ZnO NRs and the source material of Cu_3_Si NBs. We provide spatially correlated structural and optical data for individual modules of Cu-doped ZnO NRs assembled on a Cu_3_Si NB by resolving them along the different positions on the NB. Ensemble-averaged versus individual behaviors of Cu-doped ZnO NRs on Cu_3_Si NBs are then compared. We further discuss the potential impact of such ZnO-derived NRs on their relatively unexplored biological and biomedical applications. Our efforts will be particularly useful when exploiting each integrated module of self-aligned, Cu-doped ZnO NRs on a NB as a discretely addressable, active element in solid-state sensors and miniaturized luminescent bioprobes.

## 1. Introduction

Optical properties of one dimensional (1D) zinc oxide nanowires and nanorods (ZnO NWs and NRs) have been extensively studied and optimized for photonic [1,2,3,4], optoelectronic [5,6,7,8,9,10,11], and biosensing applications [12,13,14]. One particularly effective strategy for tuning the intrinsic properties of ZnO is to introduce dopants of transition metal ions such as copper (Cu). Many studies have previously demonstrated that the incorporation of Cu into the ZnO lattice can significantly alter the structural, chemical, optical, and electrical properties of ZnO [15,16,17,18,19,20,21,22,23,24,25,26,27,28]. These endeavors have been largely focused on thin film and powder structures of ZnO [15,16,17,18,19,20]. For instance, Cu doping into ZnO thin films can reduce their bandgaps [15] and induce ferromagnetic behaviors unlike neat ZnO or Cu [17,18]. In other examples, Cu doping of ZnO can produce highly nonlinear current–voltage characteristics used in varistors [19] and, together with Ga, Cu-incorporated ZnO thin films have been constructed as cathodoluminescent screens [20].

More recently, some efforts have been made to produce 1D forms of Cu-doped ZnO. They have been produced via hydrothermal [21], chemical vapor deposition (CVD) [22,23,24,25,26], thermal diffusion [27], and electrochemical deposition [28] methods. Among these, CVD has often been the method of choice, since the gas phase approach can minimize unwanted incorporation of chemical impurities during synthesis that may otherwise arise from the presence of counter ions and bystander species in solutions. In previous CVD approaches, reactions were carried out by using source mixtures of Cu/Zn, CuO/ZnO, CuI/ZnI_2_, Cu(C_5_H_7_O_2_)_2_/Zn/(Zn(C_2_H_3_O_2_)_2_·2H_2_O)_2_, or Cu film coating ZnO [22,23,24,25,26]. Regardless of the dimensionality of the synthesized materials, the development of Cu-doped ZnO has so far been geared towards magnetic, electrical, and optoelectronic functions to create better luminescence activators, dilute magnetic semiconductors, and multispectral photodetectors. For reasons, Cu-doped ZnO in previous studies has been largely examined for its properties associated with ferromagnetism, near-band-edge-related photoluminescence, and photon-induced current–voltage relationships.

We have previously demonstrated that ZnO NRs can be successfully employed in bioanalyte detection [12,13,14,29,30,31,32,33]. In these efforts, pristine ZnO NRs served as efficient subwavelength waveguides and surface evanescent wave carriers, which subsequently permitted ultrasensitive detection of DNA- and protein-derived fluorescence signals. Being utilized as passive waveguides of non-intrinsic light generated by fluorophore-conjugated biomolecules, ZnO NRs in these applications were designed to be optical quality single crystals, free of chemical and crystalline defects. The undoped ZnO NRs exhibited optical transparency in the visible range needed in fluorescence-based bioassays with no absorption or emission under commonly used visible excitation settings. However, rather than simply utilizing them as passive components, transforming ZnO NRs into active waveguides of visible light by augmenting their optical properties can be highly beneficial in biodetection. Exemplar uses of active visible light waveguides include in vivo bioprobes, luminescent markers, and local light sources in integrated optical biosensors.

In this study, we devise a straightforward and effective method to synthesize Cu-doped ZnO NRs using a novel multifunctional platform of copper silicide nanoblocks (Cu_3_Si NBs). The Cu_3_Si NBs play multiple roles, serving as catalysts for the ZnO NR growth, a local Cu source for doping the NRs, and prepatterned guides to aid the local assembly of the NRs on the growth substrate. The Cu_3_Si bases are first formed into square or rectangular nanoblocks (NBs) on Si in well-defined directions. Subsequent ZnO NR growth yields a forest of Cu-doped ZnO NRs vertically grown from the base of each Cu_3_Si NB. The resulting assembly of ZnO NRs on the Cu_3_Si NB resembles thick bristles on a brush head. The crystalline structures, optical properties, and spectroscopic signatures of the resulting Cu-doped ZnO NRs on the NBs are then ascertained at both the ensemble and individual block levels. Among the different material characterization methods that were used, fluorescence emission and Raman scattering profiles are particularly sensitive to Cu incorporation into ZnO NRs. With the use of Cu_3_Si NBs as an effective Cu dopant source, we also show that the optical properties of the NRs are significantly altered from pristine ZnO materials. ZnO NRs synthesized on Cu_3_Si NBs emit strong fluorescence in the visible range that is not seen from either Cu_3_Si NBs or undoped ZnO NRs. Cu-doped ZnO NRs also exhibit phonon modes forbidden by the Raman selection rule of wurtzite ZnO and cause large peak shifts in Raman scattering. Distinct from many previous studies investigating macroscopic or ensemble-averaged behaviors of Cu-doped ZnO, we ascertain the structural and optical characteristics of Cu-doped ZnO NRs on Cu_3_Si NBs by spatially correlating fluorescence and Raman data collected from individual NBs.

## 2. Experimental Methods

Cu_3_Si NBs were first synthesized on clean Si(100) substrates (Silicon Quest International Inc., San Jose, CA, USA) in a CVD reactor. A mixture of 0.9 g of CuO and 0.6 g of graphite, obtained from Alfa Aesar (Ward Hill, MA, USA), was placed on a source boat at the center of a horizontal resistance tube furnace. A target boat containing the Si(100) substrate was placed at the downstream side, 12 cm away from the center. The temperature of the furnace was then raised to 800 °C for 1 h under a constant Ar flow of 100 standard cubic centimeters per minute (sccm). During the CVD reaction, CuO was reduced to Cu which further reacted with Si, producing Cu_3_Si nanostructures along the Si atom directions on Si(100). ZnO NRs were subsequently grown using the Cu_3_Si NBs as catalysts. The Cu_3_Si NBs/Si(100) sample was cleaned with a 1:5 mixture of NH_3_F/HF–deionized water (DI) for 25 min and then in 0.01 N HNO_3_ for 40 min. ZnO NRs were synthesized in a CVD reactor kept under 100 sccm of Ar flow, similar to the setup described above. The source and target boats contained a 1:2 mixture of ZnO (99.999%) and graphite (99%) powder (Alfa Aesar Inc., Tewksbury, MA, USA) by weight and the Cu_3_Si NBs/Si(100) sample, respectively. The CVD reaction was held at 1000–1050 °C for 4 h. Alternatively, for synthesizing undoped ZnO NRs, 20 nm Au nanoparticles (Au NPs) from Ted Pella, Inc. (Redding, CA, USA) on a Si wafer were used as catalysts. The source materials, a 1:2 mixture of ZnO to graphite powder (0.45 g total), were placed in a quartz boat at the furnace center. The furnace was heated to 950 °C for 40 min under a 100 sccm Ar flow. Upon synthesis, the growth plate contained a dense mat of undoped ZnO NRs from which the NRs were sonicated off and dispersed in ethanol. Reconstituted ZnO NRs were then deposited onto a clean Si substrate via drop casting.

The Cu_3_Si NBs as well as doped and undoped ZnO NRs were subsequently characterized using a scanning electron microscope (SEM), FEI Nova NanoSEM 450 (FEI Company, Hillsboro, OR, USA), operated at 20 keV. For all optical measurements, a Zeiss Axio Imager A2M (Carl Zeiss, Inc., Thornwood, NY, USA) microscope equipped with an AxioCAM HRm digital camera (Carl Zeiss Microscopy GmbH., Jena, Germany) was employed. Reflected bright-/dark-field illumination and unpolarized fluorescence excitation were produced using a 12 V/100 W halogen lamp (Carl Zeiss Microscopy GmbH., Jena, Germany) and a 120 W mercury vapor lamp (X-Cite 120Q, Carl Zeiss Microscopy GmbH., Jena, Germany), respectively. Various spectroscopic settings were employed in order to check fluorescence emission of the nanomaterials in the visible window. Images were acquired with EC Epiplan-NEOFLUAR 50X (numerical aperture, NA = 0.8) and 100× magnification (NA = 0.9) objective lenses using a 2 s exposure. X-ray diffraction (XRD) measurements were made using a Rigaku Ultima IV X-ray diffractometer (Rigaku Corp., Tokyo, Japan), operated with an accelerating voltage of 40 kV under Cu Kα radiation (1.542 Å, 1.5104) and scanned in the range of 2θ = 30–70° at a rate of 1°/min. Ultraviolet–visible (UV–vis) absorption spectroscopy data were collected using an Agilent 8453 UV–vis spectrometer (Agilent Tech, Santa Clara, CA, USA). Fourier transform infrared (FTIR) spectroscopy measurements were taken using an Agilent Technologies Cary 670 Spectrometer (Santa Clara, CA, USA) with a home-built attenuated total reflectance (ATR) attachment. Raman spectra and maps were acquired using a LabRAM HR Evolution Raman confocal microscope (Horiba Instruments Inc., Sunnyvale, CA, USA) with a long working distance and 100× objective lens of 0.8 NA (Olympus Corp., Waltham, MA, USA). Raman signals upon 532 nm laser excitation were collected in the wavenumber range of 50–500 cm^−1^ using an 1800 lines/mm grating and a charge-coupled-device detector.

## 3. Results and Discussion

**Figure 1:**
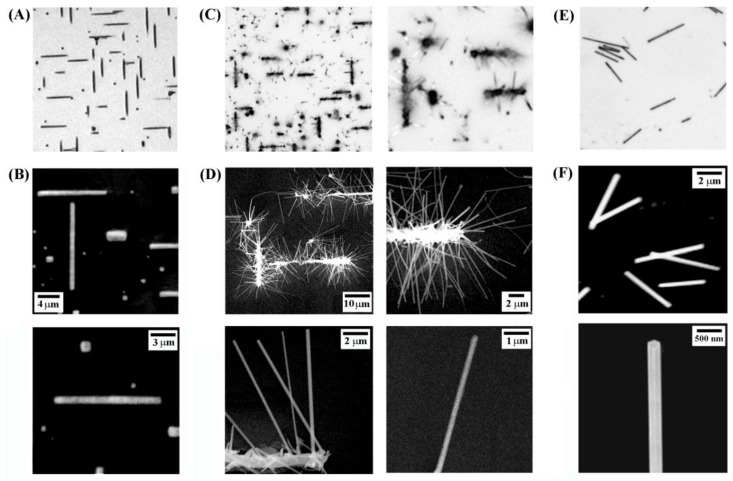
Bright-field and SEM images showing the structures of Cu_3_Si nanoblock (NB) catalysts, ZnO nanorods (NRs) grown on Cu_3_Si NBs (Cu-doped ZnO NRs), and ZnO NRs grown on Au nanoparticles (NPs) (undoped ZnO NRs). (**A**,**B**) As-synthesized Cu_3_Si NBs on Si. Upon CVD, Cu_3_Si NBs nucleate and self-assemble into NBs on the Si(100) growth substrate, as shown in the optical (**A**) and SEM (**B**) panels; (**C**,**D**) As-synthesized Cu_3_Si NBs on Si such as the ones displayed in (**A**,**B**) were used as catalysts to directly grow ZnO NRs on the NBs. Cu-doped ZnO NRs can be successfully produced on Cu_3_Si NBs, seen as brushlike structures in the bright-field image in (**C**); The wire-like shape of the Cu-doped ZnO NRs extended from a Cu_3_Si NB base can be clearly seen in the zoomed-in SEM panels in (**D**); (**E**,**F**) The optical and SEM panels in (**E**,**F**), respectively, are typical images of undoped ZnO NRs produced by using Au NP catalysts. The second row of panels shown in (**B**–**F**) displays zoomed-in views of the nanostructures shown in the first row images.

The bright-field optical image in Figure 1A and SEM panels in Figure 1B display low- and high-magnification views, respectively, of the as-synthesized Cu_3_Si NBs. The Cu_3_Si NBs on Si(100) were aligned in two specific crystal directions along [011] and [01-1] as previously reported [34,35], consistent with the underlying Si atom arrangement. The Cu_3_Si NBs shown in Figure 1 are approximately 750–800 nm in diameter and 11–14 μm in length. The Cu_3_Si NBs typically exhibit an aspect ratio much greater than 1:10. In addition, square-shaped Cu_3_Si nanocrystallites (NCs) can also be seen in Figure 1A,B. The small Cu_3_Si NCs are the dominant form on samples grown only for a short time (5–10 min), making them early-stage structures before developing into the highly elongated constructs of NBs. With increasing growth time, the NCs become lengthened along either [011] or [01-1] on the substrate of Si(100), sometimes merging with other NBs growing nearby.

Figure 1C,D display the bright-field and SEM images of the ZnO NRs grown on the Cu_3_Si NBs. The panels clearly show that the resulting NR growths occurred on Cu_3_Si NBs but not in other areas on the substrate. Thus, Cu_3_Si NBs can serve as effective catalysts for the nucleation and growth of the ZnO NRs. The as-grown structures of ZnO NRs jutting out of each Cu_3_Si NB resemble dense bristles on a brush head. The assembled structures of multiple modules of NRs/NB as well as a single module of NRs/NB can be clearly seen in the zoomed-in SEM panels in Figure 1D. The NRs shown in Figure 1D are approximately 160–300 nm in diameter and 10–12 μm in length, grown vertically from the NB base with one end free and the other end attached to the Cu_3_Si NB. Figure 1E,F show the bright-field and SEM images of undoped ZnO NRs. The undoped NRs were reconstituted and redeposited onto a clean Si from the growth plate containing a dense mat of NRs. The basal (hexagonal end) and prismic (rectangular side) facets of the pristine ZnO NR crystals can be clearly seen in the high-magnification SEM image in Figure 1F.

**Figure 2:**
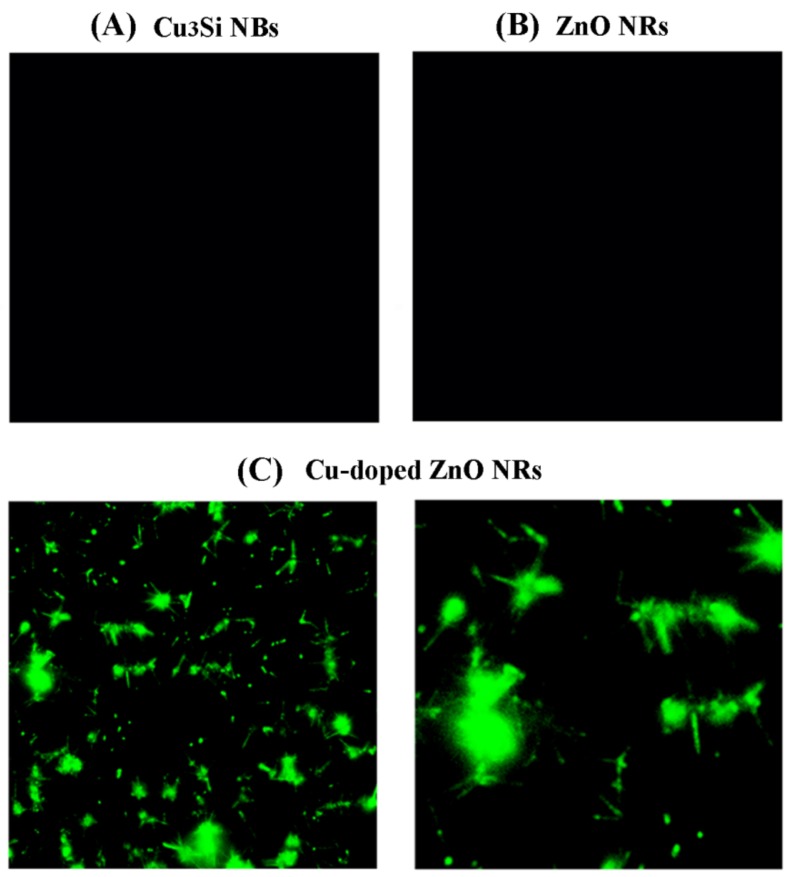
Fluorescence panels of the Cu_3_Si NB, Cu-doped ZnO NRs on Cu_3_Si NB, and neat ZnO NR samples shown in Figure 1. No fluorescence emission was seen in the visible range from the samples containing (**A**) Cu_3_Si NBs and (**B**) undoped ZnO NRs; (**C**) In contrast, strong fluorescence from the samples of ZnO NRs grown on Cu_3_Si NBs was observed due to the doping of ZnO NRs with Cu from Cu_3_Si NB catalysts. The fluorescence images in (**A**,**B**) are 300 μm × 300 μm in size. The left and right panels shown in (**C**) are 160 μm × 160 μm and 50 μm × 50 μm, respectively.

After examining the physical dimensions and morphologies of the nanostructures, we then investigated the optical properties of the undoped and Cu-doped ZnO NR samples. ZnO NRs synthesized using Au NPs versus Cu_3_Si NBs exhibited vastly different fluorescence emission behaviors under visible excitation. Figure 2A,B display representative fluorescence panels collected from the samples of Cu_3_Si NBs alone and neat ZnO NRs grown on Au NPs, respectively. Excitation and collection wavelength ranges (λ_ex_/λ_col_) used were 450–490/540–552 and 510–540/575–640, all in units of nm. Cu_3_Si NBs and undoped ZnO NRs did not show any fluorescence under these settings. Contrastingly, as-grown ZnO NRs produced on Cu_3_Si NBs yielded strong, stable fluorescence in the specified emission wavelength range as displayed in Figure 2C, which was due to Cu doping into the NRs. Fluorescence from vertically grown NR ensembles yielded furry green patches that span along the nonemitting Cu_3_Si NBs. The hazy fluorescence pattern is due to the collective emission of many m-long doped ZnO NRs vertically straddling the focal plane. In contrast, those NRs lying in the imaging plane unmistakably appear as fluorescing rods in the same fluorescence images. Similar fluorescence emission was also confirmed in the red range for the ZnO NRs grown on Cu_3_Si NBs.

**Figure 3:**
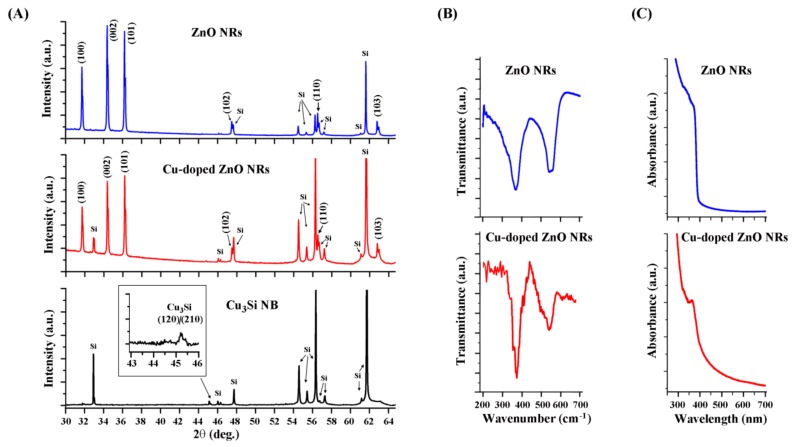
Diffraction and spectroscopic data of the NBs and NRs. (**A**) XRD data of Cu_3_Si NBs (black), Cu-doped ZnO NRs on Cu_3_Si NBs (red), and undoped ZnO NRs (blue); (**B**) ATR-FTIR spectra of undoped (blue) and Cu-doped (red) ZnO NRs; (**C**) UV–vis spectra of undoped (blue) and Cu-doped (red) ZnO NRs.

We subsequently employed various diffraction and spectroscopic techniques to examine the different nanomaterials. Figure 3A displays XRD data of pristine ZnO NRs, Cu-doped ZnO NRs, and Cu_3_Si NBs as grown on Si wafers. The topmost spectrum shown in Figure 3A, corresponding to as-synthesized, undoped ZnO NRs, agrees well with the characteristic diffraction peaks of wurtzite ZnO crystals. The peaks at 2θ = 31.8°, 34.5°, 36.5°, 47.5°, 56.5°, and 63° belong to (110), (002), (101), (102), (110), and (103) of the ZnO NR crystal, respectively, and the unassigned peaks in the spectrum belong to the Si substrate. The middle spectrum in Figure 3A is taken from Cu-doped ZnO NRs grown on Cu_3_Si NBs, showing the same diffraction patterns as undoped ZnO NRs. We did not observe any significant deviations in terms of new peaks or peak shifts in the undoped versus Cu-doped ZnO NR samples. No peaks related to Cu or CuO appeared and only the overall peak intensities of the Cu-doped ZnO NRs decreased. This was due to the lower amounts of ZnO NRs synthesized as they form only on the Cu_3_Si NBs areas. The absence of CuO phase in the spectra indicates that the incorporated Cu level in our NR samples has not exceeded the solid solubility of Cu in ZnO which is ~1 atomic percent [36,37]. The bottommost diffraction panel in Figure 3A is from Cu_3_Si NBs self-assembled along the two preferential growth directions on Si(100). The peak appearing at 45.5° is indicative of Cu_3_Si (120)/(210) and the other peaks present are from Si [34,35].

Data in Figure 3B,C display the IR and UV–vis spectra, respectively, of undoped and Cu-doped ZnO NRs. Relatively broad peaks centered at 350 and 550 cm^−1^ in the fingerprint region of ZnO were found in the ATR FTIR spectra of both undoped and Cu-doped ZnO NRs in Figure 3B. These peaks are associated with Zn–O stretching [38,39,40]. No additional absorption peaks were identified from the Cu-doped ZnO NRs. Unlike the XRD and ATR FTIR outcomes, the UV–vis absorption profiles characterized from the two samples showed a small change in the 400–500 nm range, noticeable in Figure 3C. The absorption profile of neat ZnO NRs center in the UV region and show a sharp decrease at 390 nm. In contrast, the decrease in this region becomes more gradual and extends towards the visible range in the absorption spectrum for the Cu-doped ZnO NRs. The increased absorbance into the visible region of the Cu-doped ZnO NR sample coincides with the appearance of visible fluorescence emission from the NRs in Figure 2.

**Figure 4:**
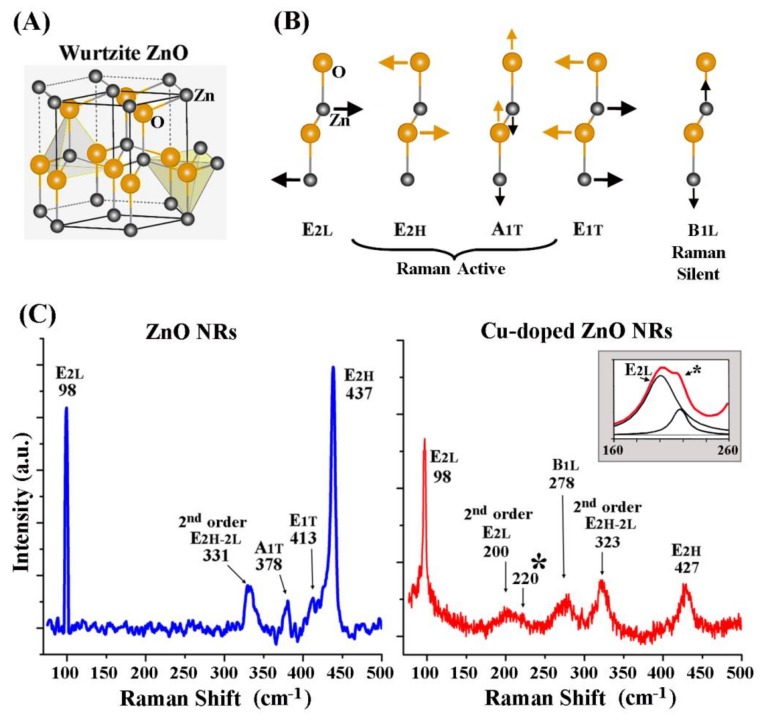
Raman spectra of undoped and Cu-doped ZnO NRs, as synthesized on Si substrates using Au NP and Cu_3_Si NB catalysts, respectively. (**A**) A ball-and-stick model of atomic arrangements of Zn and O is displayed for an undoped, wurtzite ZnO crystal; (**B**) Characteristic Raman vibration modes of wurtzite ZnO are schematically depicted; (**C**) The differences in the Raman scattering behaviors between the undoped (blue) and Cu-doped (red) ZnO NR samples are clearly resolved. The Raman spectra are ensemble-averaged signals acquired from a collection of undoped ZnO NRs (blue) and concentrated modules of Cu-doped ZnO NRs on NBs (red). The inset in the Cu-doped ZnO spectrum is the magnified view of the curve fit (red) and the peak fits (black) of the scattering data between 160 and 260 cm^−1^. The Cu(I)–O peak is marked with an asterisk.

**Figure 5:**
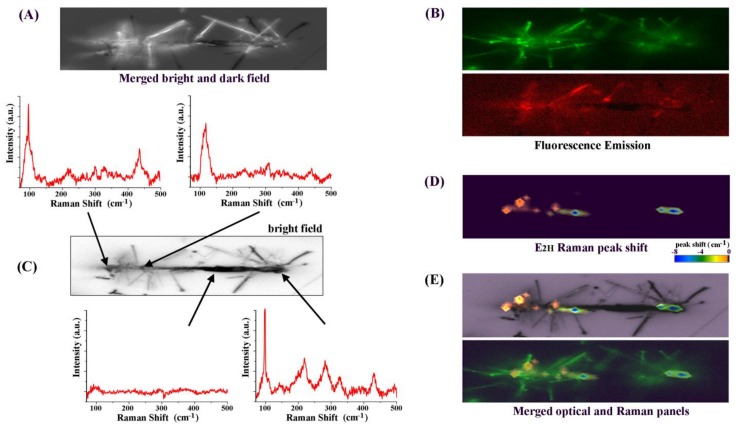
Representative panels displaying spatially correlated data between optical, fluorescence emission, and Raman scattering profiles measured from individual NBs with Cu-doped ZnO NRs. (**A**,**B**) The optical panel in (**A**) displays a superimposed, bright- and dark-field image of Cu-doped ZnO NRs on a Cu_3_Si NB. The corresponding fluorescence emission profiles along the NB are shown in panel (**B**) where the top and bottom frames display the emission collected at wavelength ranges of 510–540 nm and 575–640 nm, respectively; (**C**) Exemplar Raman spectra spatially resolved along the length of the NRs/NB module are presented; (**D**) The E_2H_ peak shift, characteristic of Cu-doped ZnO NRs grown on a Cu_3_Si NB, is mapped along the NB via NB position-resolved Raman spectroscopy; (**E**) The top and bottom panels are the merged view of bright-field and E_2H_ Raman peak shift (top) and that of fluorescence and E_2H_ Raman peak shift (bottom). All bright-field, fluorescence, and mapped Raman images shown are 4.5 μm by 20 μm in size.

Figure 4 displays the Raman scattering results from undoped and Cu-doped ZnO NRs. Wurtzite ZnO, according to group theory, has the optical modes of ***Γ****_opt_* = A_1_ + 2B_1_ + E_1_ + 2E_2_ at the point of the Brillouin zone [41,42]. A_1_, E_1_, and E_2_ are Raman active whereas B_1_ is a silent mode in pristine ZnO. The atomic arrangements of Zn and O in a wurtzite crystal structure as well as the vibrations associated with the characteristic ZnO phonon modes are depicted in Figure 4A,B. As seen from the Raman scattering data of pristine ZnO NRs in the left panel of Figure 4C, the Raman modes of E_2L_, E_2H_–E_2L_, A_1T_, E_1T_, and E_2H_ were resolved at 98, 331, 378, 413, and 437 cm^−1^, respectively. These peaks are expected from wurtzite ZnO NRs belonging to the C^4^_6v_ space group [41,42,43]. The strong peaks of high and low E_2_ reflect the chemical composition of Zn (E_2L_) and O (E_2H_) in the high-quality wurtzite ZnO NR sample. Contrarily, the Raman signals from Cu-doped ZnO NRs in the right panel of Figure 4C indicate significant deviations from the scattering behaviors of pristine ZnO NRs. Large peak shifts were observed for the E_2H_–E_2L_ and E_2H_ modes by 8–12 cm^−1^ to the left of those from pristine ZnO. In addition, the forbidden B_1L_ mode newly appeared at around 280 cm^−1^ in the Cu-doped ZnO NR Raman spectra as did the second-order E_2L_ peak centered at 196 cm^−1^. A clear indication of Cu incorporation into the Zn–O lattice was the new peak at 220 cm^−1^, which originates from the presence of Cu(I)–O in the Zn–O lattice [44,45]. The Cu(I)–O peak is marked with an asterisk in the Raman spectrum of Cu-doped ZnO NRs in Figure 4C. Although appearing small in the ensemble-averaged Raman spectra of Cu-doped ZnO NR samples, the Cu(I)–O peak was identified prominently in the Raman measurements examining single modules of NRs/NB, which will be discussed in Figure 5. Regardless, we successfully revealed additional Raman peaks and peak shifts specifically related to Cu doping of ZnO NRs, which could not be identified previously under low doping regimes. The Cu incorporation in our samples neither altered the dominant growth direction of the NRs nor changed the wurtzite crystals to an amorphous or another crystalline state, as evidenced by the XRD data in Figure 3A. However, the Cu dopant resulted in significant disruptions in the long-range order of the NR crystal, as seen by the pronounced downshift as well as the broadening and weakening of the Raman peaks. The perturbed Raman spectra of the doped relative to the pristine ZnO NR samples indicate that the symmetry of the allowed phonons is broken due to Cu introduction into the host ZnO lattice. The Cu-induced, long-range disorder in the ZnO lattice is likely to cause the contribution of *q* ≠ 0 phonons to the Raman features such as the additional second-order peaks, not observed from pristine ZnO NRs.

Our results indicated that fluorescence emission and Raman scattering were particularly sensitive to Cu addition to ZnO NRs. Hence, in addition to those collectively acquired from a group of NRs/NBs, we further examined the fluorescence emission and Raman scattering profiles of Cu-doped ZnO NRs on Cu_3_Si NBs at the individual nanostructure level. Specifically, individual NRs/NB modules were studied via spatially correlated optical, fluorescence, and Raman measurements along the length of the NB. Figure 5 displays typical, correlated data acquired by examining individual NRs/NB modules. Figure 5A is a merged bright- and dark-field image of Cu-doped ZnO NRs on a NB. Figure 5B is the corresponding fluorescence emission collected in the green (top) and red (bottom) wavelength ranges. The spectra presented in Figure 5C correspond to the spatially resolved Raman scattering signals taken from the NRs/NB module. Although overall features were similar, Raman scattering profiles from individual NRs/NB modules differed from those of NRs/NB ensembles, whose representative data are shown in Figure 4C and Figure 5C, respectively. For individual NRs/NB modules, the characteristic peaks associated with Cu doping of ZnO NRs such as the Cu(I)–O and B_1L_ modes appeared more prominently, whereas the contribution of the second-order E_2L_ mode was reduced. The location of the Cu(I)–O peak is found at 225 cm^−1^ in individual NRs/NB spectra, moved from 220 cm^−1^ for the ensemble samples. The peak shifts of the E_2H_–E_2L_ and E_2H_ modes seen in the ensemble samples were also observed in the individual NRs/NB modules.

To construct NB position-resolved Raman scattering profiles, two-dimensional (2D) Raman mapping was carried out for which a laser beam of approximately 1 µm in diameter was rastered in the vicinity of a NRs/NB module of interest. Depending on the size of the NB, the steps taken in the beam walk ranged 550–750 nm and 270–440 nm along the long and short axes of the NB, respectively. The resulting Raman map, corresponding to the E_2H_ peak shift of the NRs/NB sample in Figure 5A, is presented in Figure 5D. The two panels in Figure 5E are a superimposed view of the bright-field and Raman data (top) with that of the fluorescence and Raman data (bottom). As evidenced in the top panel of Figure 5E, the NB areas of NR growths in the optical microscopy image match with the regions of large E_2H_ peak shifts in the Raman map. As observed in the bottom panel of Figure 5E, the NB areas of large fluorescence and Raman peak shift signals are co-located. As both the visible fluorescence and the Raman peak shift are indicative of Cu doping, the correlated data set further confirms that the resulting ZnO NRs on the Cu_3_Si NB are indeed doped with Cu.

The inherent property of neat ZnO NRs showing optical transparency in the visible range has so far limited their applications to luminescence and active guiding in the spectral region of UV. Even for the limited cases employing ZnO NRs as passive waveguides in the visible region, the endeavors were largely in optoelectronic fields [1,2,6,46]. Yet, these ZnO NRs and their derivatives have potential to be engineered into biodetection modules [47,48,49,50,51] for measuring protein activities and cell differentiation/growth using visible light. For such biological applications, visualization of the NRs is essential because determining the exact placement and distribution of the NRs in tissues and cells/cellular components is critical for interpreting results. Our efforts demonstrated in this study may be significant in this regard. For instance, the visible luminescence from vertically grown Cu-doped ZnO NRs on NBs that are self-aligned laterally on a Si wafer may be used in the streamlined fabrication of nanophotonic circuitries in integrated biosensors for bioanalyte detection via fluorescence resonance energy transfer [52,53] and fluorescence anisotropy [54,55]. The intense green fluorescence produced by the Cu-doped ZnO NRs on Cu_3_Si NBs may enable precise determination of their location once coupled with cellular systems. In addition, our approach demonstrated in this paper can greatly increase the versatility of ZnO-derived NRs in fluorescence-based biodetection by promoting them to effectively function as an active, not just passive, guiding element for visible light. For example, highly controlled spatial delivery of the green light from the Cu-doped ZnO NRs can be enabled by actively waveguiding the inherent light to the NR termini and, subsequently, to the bioanalytes of interest for exclusive excitation, without affecting nearby species whose unwanted excitation can contribute to background interference. Therefore, our efforts can be highly valuable in paving the way for new applications of ZnO-derived NRs in basic biological research and in biomedical detection which is relatively unexplored so far, particularly for the development of integrated optical biosensors and miniaturized bioprobes.

## 4. Summary

In summary, we have demonstrated a straightforward and effective method to synthesize Cu-doped ZnO NRs using a novel system of Cu_3_Si NBs pre-aligned laterally on a Si wafer. We showed that the unique platform of Cu_3_Si NBs can play multifunctional roles in the CVD synthesis. They functioned as a catalyst for ZnO NR growth, a local Cu dopant source, and a pre-assembled guide for NR assembly on the Si substrate. As a result, dense, bristle-like, Cu-doped ZnO NRs were nucleated and vertically grown from each Cu_3_Si NB base. We subsequently characterized their structural, optical, and spectroscopic characteristics at both the ensemble and individual nanostructure levels. In particular, we have successfully performed spatially correlated structural and optical measurements at the level of each NRs/NB module, including NB position-resolved fluorescence and Raman spectra. We have ascertained the augmented optical properties of doped ZnO NRs relative to those of pristine ZnO NRs and Cu_3_Si NBs, and further discussed their potential biodetection applications. Specifically, Cu-doped ZnO NRs showed an extended absorption edge and strong luminescence in the visible range. The inclusion of Cu into ZnO NR crystals induced the emergence of the forbidden B_1L_ and the second-order E_2L_ modes as well as considerable peak downshifts for the E_2H_–E_2L_ and E_2H_ phonon modes in Raman spectra. Coupled with the facile assembly of inherently fluorescing NRs on the bases of self-aligned NBs and the widespread use of fluorescence in solid-state biodetection, our Cu-doped ZnO NRs/NB architectures may be particularly advantageous for use in active visible waveguiding, nanobiophotonics, bioprobes, and luminescent markers.
